# Stem and Progenitor Cells for Musculoskeletal Disease Modeling and Tissue Repair

**DOI:** 10.3390/bioengineering11121175

**Published:** 2024-11-21

**Authors:** Songlin He, Wanting Niu, Zhong Alan Li

**Affiliations:** 1 Department of Biomedical Engineering, The Chinese University of Hong Kong, NT, Hong Kong SAR 999077, China; songlinhe@link.cuhk.edu.hk; 2 Center for Neuromusculoskeletal Restorative Medicine, Hong Kong Science Park, NT, Hong Kong SAR 999077, China; 3 Tissue Engineering Labs, VA Boston Healthcare System & Department of Orthopedics, Brigham and Women’s Hospital, Harvard Medical School, Boston, MA 02115, USA; wantingniubioe@gmail.com; 4 School of Biomedical Sciences, The Chinese University of Hong Kong, NT, Hong Kong SAR 999077, China; 5 Institute for Tissue Engineering and Regenerative Medicine, The Chinese University of Hong Kong, Shatin, Hong Kong SAR 999077, China; 6 Shun Hing Institute of Advanced Engineering, The Chinese University of Hong Kong, NT, Hong Kong SAR 999077, China

Musculoskeletal conditions such as osteoarthritis (OA), bone fracture, and sarcopenia are highly prevalent [1]. As aging is a prominent risk factor for many musculoskeletal diseases, the socioeconomic burden of musculoskeletal disorders is expected to increase due to the aging of the world’s population [2]. Stem and progenitor cells play a central role in the regeneration of bone, cartilage, skeletal muscle, tendon, and ligament. Research into promoting stem cell proliferation and directed differentiation is crucial for understanding stem cell fate decisions and accelerating their clinical applications. Different types of stem cells have been investigated for their potential applications in musculoskeletal regeneration. However, challenges remain in guiding stem cell differentiation with effective biochemical and biophysical signals to restore musculoskeletal tissue functions. Next-generation tissue engineering strategies require the precise regulation of stem cell behaviors to unleash their therapeutic potential and achieve optimal patient outcomes.

The clinical translation of biomedical technologies relies heavily on effective disease models. Preclinical studies typically begin with in vitro tests in 2D or 3D cell cultures or with tissue explants, which are followed by in vivo evaluation with animal models. Conventional cell cultures are mostly oversimplified and fail to faithfully recapitulate the physiology and pathology of native musculoskeletal tissues. Human tissue explants as disease models suffer from low availability, cell death at tissue edges, and varying tissue properties depending on the harvest site. Animal models have been widely utilized in musculoskeletal research, but their predictive power in therapeutic testing has long been questioned, primarily because of their differences in comparison to human patients in the genetic background, as well as the anatomy and biomechanics of the musculoskeletal system. Human cell-based advanced in vitro models, particularly organoids and organ-on-a-chip (OoC) systems, are gaining increasing attention. Stem/progenitor cells can be harvested from different tissue sources, self-renew, and differentiate into various musculoskeletal lineages to recapitulate different native tissues [3]. Thus, these cells are among the most commonly used cell types for establishing organoids and OoCs to emulate native musculoskeletal tissue and organ systems under physiological and pathological conditions. Human stem/progenitor cell-based organoids and OoCs hold the potential to achieve a paradigm shift in mechanistic studies on musculoskeletal conditions, as well as the evaluation of potential therapeutic options.

This Special Issue is dedicated to exploring strategies for musculoskeletal disease modeling and tissue repair with human stem/progenitor cells and includes three original articles and three review papers. The three review articles provide a broad overview of strategies for tissue regeneration from the perspectives of cell metabolism regulation, cell gene editing, and intercellular communication. The review article by Franco-Obregón and colleagues provides an overview of the metabolic patterns and plasticity of muscle cells and the effects of magnetic field therapy on muscle cell fate [4]. This review summarizes the metabolic and phenotypic characteristics of glycolytic and oxidative muscle in response to exercise and magnetic fields, as well as the effects of endurance exercise on the crosstalk between oxidative muscle and adipose tissue. The authors further explained the Ca^2+^ signal transduction in oxidative muscle development and the joint influence of magnetic field and endurance training. They noted that the reddening–browning cycle of magnetically induced oxidative muscle development is closely related to metabolic adaptations based on endurance exercise. Finally, they compared the relationship between extremely low frequency pulsed electromagnetic fields and both resistance and endurance training, and pointed out the potential of magnetic mitosis in guiding cell fate and tissue regeneration.

The review article by Krut and coworkers focuses on ultrasound-guided gene delivery methods [5]. The authors discussed the application of ultrasound in tissue regeneration and systematically summarized the preclinical studies on tissue regeneration using ultrasound. The article explained the principles of ultrasound-based gene delivery and summarized the application of ultrasound-based technology in bone tissue regeneration, myocardial ischemia treatment, peripheral ischemia therapies, and pancreatic islet regeneration. The authors further elaborated on the potential of ultrasound technology in other tissue engineering and regeneration applications. Finally, the authors summarized the clinical application of ultrasound technology and its potential risks and provided a potential gene delivery strategy.

The review by Fan and colleagues explores the role of exosomes in the pathogenesis, progression, and treatment of OA [6]. The authors described the biogenesis of exosomes and different exosome sources. It was pointed out that exosomes from different tissues have specific regulatory effects on the source tissues, and exosomes from joint tissues, including the MSCs present in these tissues, have a regulatory effect on OA. The authors further summarized the strategies for exosome extraction, bioengineering modification, and delivery, as well as describing the in vivo efficacy of different exosome-based OA treatment methods. The authors finally provided an outlook on exosomes for OA diagnosis and treatment.

In the research article by Nadra and colleagues, a platelet-rich plasma lysate gelatin hydrogel was constructed for bone repair [7]. Inspired by the healing mechanism of blood clots, the authors constructed gelatin hydrogels using a conjugate of gelatin and hydroxyphenyl propionic acid, and evaluated the effects of platelet-rich plasma (PRP) lysate and PDGF-BB on MSC osteogenic differentiation. This study showed that PRP lysate can effectively promote the migration of MSCs and stimulate the osteogenic differentiation of MSCs after being incorporated into the gel. Further, the hydrogel could be embedded in a porous calcium phosphate block to promote bone repair.

In the research article by Yan and coworkers, meniscus samples were collected from rabbits with meniscus tears at different time points after surgery to measure the apoptosis index and matrix degeneration of the tissue [8]. The authors observed more prominent apoptosis in the degenerated area of the meniscus, and the apoptotic index reached the highest level one week after meniscus tear. The authors proposed a potential strategy for meniscus repair by mitigating meniscus cell apoptosis. They effectively promoted meniscus repair by targeting caspase inhibitors with silica nanoparticles and fibrin gel.

In the research article by Taninak and collaborators, the protection of degenerated articular cartilage was attempted via the use of adipose-derived stem cell (ADSC) sheets [9]. The authors induced the chondrogenic differentiation of ADSCs with PRP and subsequently treated the cells with ascorbic acid to form a sheet-like structure. They then transplanted the cell sheets to the articular surface in a rabbit OA model established via anterior cruciate ligament transection. The results showed that the cell sheets could effectively reduce inflammatory responses and secrete growth factors with cartilage-protective properties, inhibiting the progression of articular cartilage degeneration.

The six articles in this Special Issue focused on the regulation, characterization, and applications of stem/progenitor cells, particularly mesenchymal stem cells, for the effective treatment of musculoskeletal diseases. This Special Issue provides comprehensive insights into biomaterials, scaffolds, and cutting-edge manufacturing and delivery technologies designed to modulate stem cell behaviors; it presents several promising approaches aimed at promoting tissue regeneration, maintaining tissue homeostasis, and mitigating musculoskeletal degeneration. Future research in these rapidly evolving research areas is expected to facilitate the clinical translation of stem/progenitor cell technologies and lead to improved treatment outcomes for patients suffering from various musculoskeletal conditions.
